# Unveiling the antioxidant and anti-inflammatory potential of syringic acid: mechanistic insights and pathway interactions

**DOI:** 10.3389/fphar.2025.1615294

**Published:** 2025-07-09

**Authors:** Zhejun Zhao, Qiuhua Yang, Yuanlong Sun, Xiaofen Ruan

**Affiliations:** ^1^ Cardiovascular Research Institute of Traditional Chinese Medicine, Shuguang Hospital of Shanghai University of Traditional Chinese Medicine, Shanghai, China; ^2^ Cardiovascular Department, Shuguang Hospital of Shanghai University of Traditional Chinese Medicine, Shanghai, China; ^3^ Department of Pharmacological Sciences, State University of New York at Stony Brook, Stony Brook, NY, United States

**Keywords:** syringic acid (PubChem CID 10742), oxidative stress, inflammation, pharmacological activity, pharmacokinetics, toxicity

## Abstract

**Background:**

Syringic acid (SA), a naturally occurring phenolic acid, has garnered significant attention for its antioxidant and anti-inflammatory properties. However, the mechanisms underlying these effects and their potential therapeutic applications require further elucidation.

**Methods:**

A comprehensive literature review was conducted using PubMed and Web of Science (1965–2024) to investigate the antioxidant and anti-inflammatory mechanisms of SA, with a focus on oxidative stress and inflammatory pathways. For insights related to traditional Chinese medicine (TCM), we referenced Chinese literature. Articles focusing on agriculture, industry, and economics are excluded.

**Results:**

SA exerts potent antioxidant and anti-inflammatory activities through multiple mechanisms. Specifically, it mitigates OS by scavenging free radicals, enhancing endogenous antioxidant defenses, and activating the KEAP1/NRF2 pathway. It also inhibits inflammation by downregulating key mediators, including NF-κB, TLR4, HMGB1, MyD88, and TRAF6. Crosstalk between NRF2, NF-κB, and PI3K/AKT pathways reveals SA’s involvement in cellular pathophysiological processes such as apoptosis, ferroptosis, and endoplasmic reticulum stress.

**Conclusion:**

SA’s robust antioxidant and anti-inflammatory mechanisms underscore its promise as a therapeutic agent. Future research should address its pharmacokinetics, safety profile, and clinical potential.

## 1 Introduction

Natural metabolites from plants are a rich source for drug discovery, with polyphenols serving as potent antioxidants found abundantly in fruits and vegetables (de Araújo et al., 2021). Among them, phenolic metabolites-comprising tannins, flavonoids, phenolic acids, and lignans-are widely known for their antioxidant, anti-inflammatory, antimicrobial, and anticancer activities (Fu et al., 2022; Milla and Kerpper, 2023). One of the most well-studied phenolic acids is gallic acid (GA), recognized for its strong antioxidant activity and therapeutic benefits in diseases including cardiovascular diseases (Kosuru et al., 2018), cancer (Ashrafizadeh et al., 2021), and diabetes mellitus (Xu, Y. et al., 2021).

**FIGURE 1:**
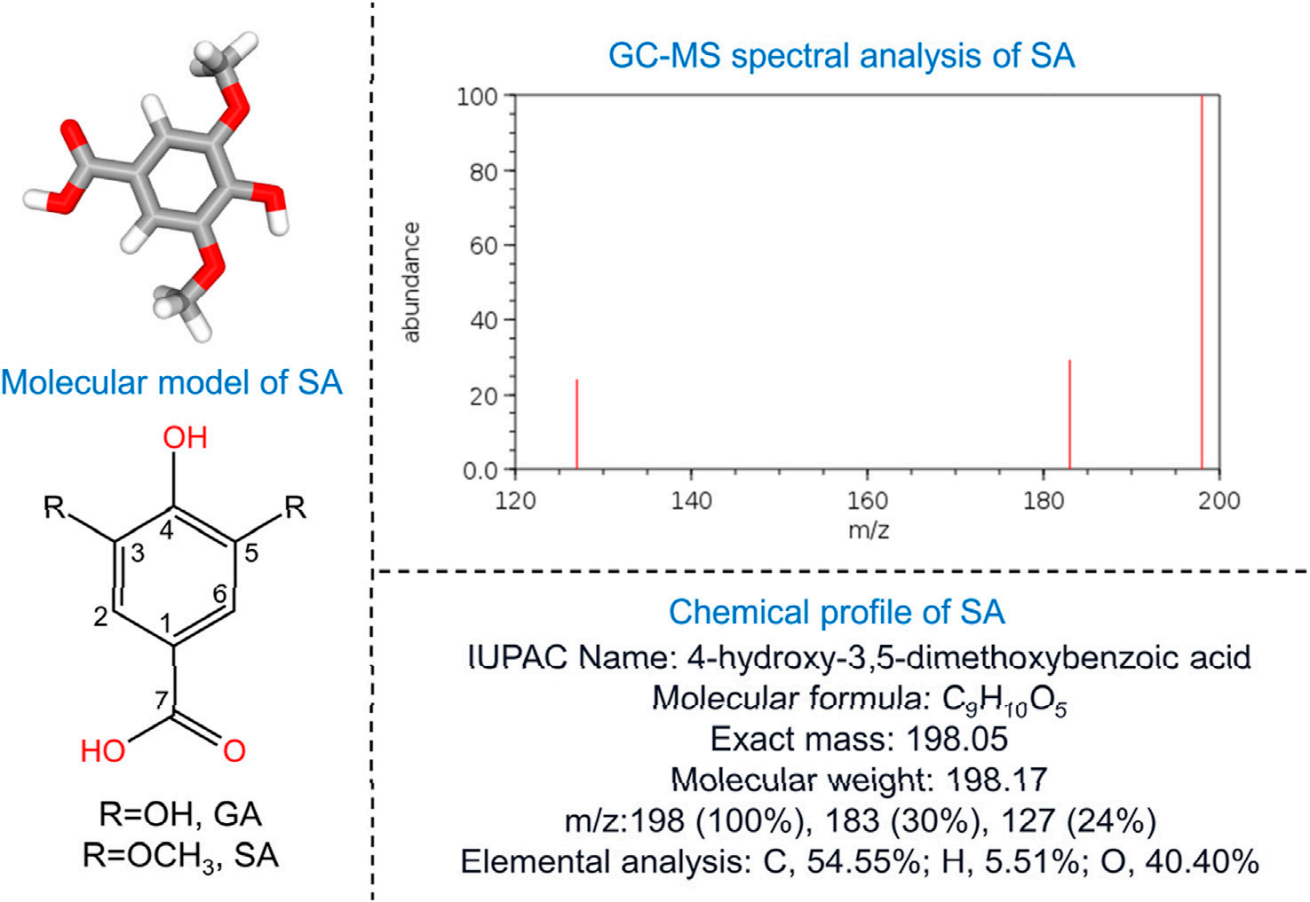
Fundamental characteristics of SA. The left side of the figure illustrates the molecular model of SA, highlighting the substitutions at the 3 and 5 carbon positions: hydroxyl groups denote GA, while methoxy groups indicate SA. The top-right corner displays the GC-MS spectral analysis of SA, and the bottom-right corner provides the chemical profile. GA, gallic acid; GC-MS, gas chromatography-mass spectrometry; SA, syringic acid.

SA, a dimethoxybenzene and 3,5-dimethoxy derivative of GA (Figure 1), has demonstrated antioxidant, anti-inflammatory, and anticancer properties, as well as protective roles against diabetes, cardiovascular diseases, and liver damage (Srinivasulu et al., 2018). First extracted through permanganate oxidation of sinapic lignin (Bland and Logan, 1965), SA is abundantly found in dietary sources such as olives, pumpkins, grapes, blueberries, and honey (Bartel et al., 2023). Additionally, it presents in some medicinal plants such as the seed of *Lepidium virginicum* L. (“Bei Tinglizi” in Chinese), the seed of *Descurainia sophia* (L.) Webb. ex Prantl (“Nan Tinglizi” in Chinese), as well as the radix of *Paeonia lactiflora* Pall. or *Paeonia veitchii* Lynch (“Chishao” in Chinese) (Hu et al., 2022; Yang, 2020). Both Tinglizi and Chishao are considered to possess antioxidant and anti-inflammatory activities (Hsieh et al., 2020; Parker et al., 2016). Notably, wine-processed Chishao, used for treating dysmenorrhea, exhibits an increased SA content after fermentation, enhancing its anti-inflammatory effects through prostaglandin F2α inhibition, similar to non-steroidal anti-inflammatory drugs (NSAIDs) (Ferries-Rowe et al., 2020).

While comprehensive reviews have addressed SA’s occurrence, biosynthesis, pharmacology, and industrial relevance, as well as its role in neurodegenerative and civilization diseases (Bartel et al., 2023; Ogut et al., 2022; Srinivasulu et al., 2018), the precise mechanisms underlying its antioxidant and anti-inflammatory effects remain unclear. This review aims to elucidate these mechanisms by examining SA’s structure-activity relationships and its complex regulation of NRF2 and NF-κB, providing insights into its direct molecular targets. Additionally, we propose a hypothesis to explain SA’s dual pro- and anti-apoptotic roles in cancer. The review further evaluates SA’s activity on oxidative stress and inflammation-related cellular pathologies, while assessing its pharmacokinetics, safety, and therapeutic potential.

## 2 Search strategy

A comprehensive literature review was conducted using PubMed and Web of Science (1965–2024) to investigate the antioxidant and anti-inflammatory mechanisms of SA, with a focus on oxidative stress OS and inflammatory pathways. The search strategy incorporated keywords like “syringic acid,” “oxidative stress,” and “inflammation.” A total of 1,226 articles were identified. Titles and abstracts were screened for relevance, and full texts were assessed according to PRISMA (Preferred Reporting Items for Systematic Reviews and Meta-Analyses) guidelines. Inclusion criteria focused on studies exploring the pharmacological mechanisms of SA in relation to oxidative stress and inflammation, while exclusion criteria omitted articles centered on agriculture, industry, or economics. *In vitro* studies were only included if they were clearly linked to or built upon *in vivo* findings and used primarily for mechanistic exploration. Purely *in vitro* studies without any *in vivo* validation were excluded. Chinese literature was referenced for insights related to Traditional Chinese Medicine (TCM). Data extraction prioritized outcomes addressing SA’s molecular targets, pathways, and therapeutic potential. Two independent reviewers conducted the selection and quality assessment processes, evaluating study design, sample size, methodology, and risk of bias, with discrepancies resolved through discussion. Articles containing suspicious data errors, omissions, or duplications were excluded to ensure the integrity and reliability of the analysis.

## 3 Antioxidant mechanisms of SA

Living cells constantly encounter reactive oxidative molecules from both externally and internally sources. These species, including free radicals or non-radical forms like H_2_O_2_, readily capture electrons from surrounding molecules, initiating chain reactions that can damage cellular structures (Filomeni et al., 2015). Under physiological conditions, cells tightly regulate the production of free radicals, including reactive oxygen species (ROS) and reactive nitrogen species (RNS), to maintain redox homeostasis through intrinsic antioxidant defense system (Meulmeester et al., 2022). They are predominantly generated within the mitochondrial electron transport chain, particularly at complexes I and III, where electron leakage facilities the partial reduction of molecular oxygen, leading to the formation of superoxide radicals (Dan Dunn et al., 2015). These superoxide radicals are rapidly converted into the less reactive H_2_O_2_ through the catalytic action of superoxide dismutase (SOD). However, under conditions such as elevated flux through the electron transport chain, reduced mitochondrial efficiency, or compromised endogenous antioxidant defense, this redox equilibrium becomes disrupted (Dan Dunn et al., 2015). This imbalance leads to oxidative stress, wherein excess ROS are inadequately neutralized, causing damage on cellular structures and functions. This review explores the mechanisms by which SA mitigates oxidative stress through four key aspects: structural activity, modulation of free radical production, enhancement of the endogenous antioxidant defense system, and activation of molecular signaling pathways. The effects of SA on oxidative stress-related targets are summarized in Table 1.

**TABLE 1:** The effects of SA on OS-related targets in different types of experiments.

Species and strain	Model or disease	Tested dosage (duration)	Positive control	Negative control	Administration approach	Target	References
Rat (Sprague-Dawley)	Testicular damage induced by lead acetate	25 and 50 mg/kg (7 days)	—	Saline	Gavage	↓8-OHdG, ↓MDA, ↑CAT, ↑GPx, ↑GSH, ↑NQO1, ↑NRF2, ↑SOD	Akarsu et al. (2023)
	Testicular injury induced by I/R	50 and 100 mg/kg (2.5 h)	—	DMSO	Intraperitoneal injection	↓8-OHdG, ↓MDA, ↑CAT, ↑SOD, ↑TAS, ↓TOS	Demir et al. (2023)
	Cerebral ischemia induced by middle cerebral artery occlusion	8 and 10 mg/kg (6 and 24 h)	—	—	Intraperitoneal injection	↓MDA, ↑SOD	Güven et al. (2015)
	Ovarian injury induced by cisplatin	5 and 10 mg/kg (3 days)	—	DMSO	Intraperitoneal injection	↓4-HNE, ↓TOS, ↑GPx, ↑GSH, ↑HO-1, ↑NRF2, ↑SOD, ↑TAS	Demir (2023)
	Diabetic nephropathy induced by streptozotocin	25 and 50 mg/kg (4 w)	—	—	Gavage	↓MDA, ↑CAT, ↑GSH, ↑GPx, ↑NQO1, ↑NRF2, ↑SOD	Sherkhane et al. (2023)
	Sciatic nerves I/R	10 mg/kg (24 h)	Methylprednisolone	Saline	Intraperitoneal injection	↓MDA, ↑SOD	Tokmak et al. (2017)
	Spinal cord I/R	10 mg/kg (24 h)	Methylprednisolone	Saline	Intraperitoneal injection	↓MDA, ↑SOD	Tokmak et al. (2015)
	Myocardial infarction induced by isoproterenol	12.5, 25, and 50 mg/kg (21 days)	—	Saline	Gavage	↓MDA, ↑CAT, ↑GPx, ↑GSH, ↑GST, ↑SOD, ↑TAC	Shahzad et al. (2019)
Rat (Wistar)	Hepatic encephalopathy induced by thioacetamide	50 and 100 mg/kg (14 days)	—	—	Gavage	↓8-OHdG, ↓iNOS, ↓MDA, ↓ROS production, ↑SOD, ↑GSH	Ferah Okkay et al. (2022)
	Ulcerative colitis induced by acetic acid	10, 25, and 50 mg/kg (5 days)	Dexamethasone	Saline	Intra-rectally	↑HO-1, ↑NQO1, ↑NRF2	Ekhtiar et al. (2023)
	Lung fibrotic induced by NDMA	50 mg/kg (28 days)	Ascorbic acid	Saline	Gavage	↓MDA, ↑CAT, ↑GPx, ↑GSH, ↑GST, ↑SOD, ↑NRF2	Somade et al. (2022)
	Hypertension induced by L-NAME	25, 50, and 100 mg/kg (28 days)	—	Saline	Gavage	↑CAT, ↑GPx, ↑GSH, ↑SOD, ↑Vitamin C, ↑Vitamin E	Kumar et al. (2012)
	Autism induced by valproic acid	25, 50, and 100 mg/kg (28 days)	—	Saline	Intraperitoneal injection	↓MDA, ↑GSH, ↑CAT	Mallan and Singh (2023)
	Hippocampal tissue damages induced by sub-chronic deltamethrin exposure	25 mg/kg (60 days)	—	Corn oil	Gavage	↓ROS/RNS, ↑TAS	Ogut et al. (2019)
	Diabetic rats induced by streptozotocin	2.5% and 5% (14 days)	—	—	Topical application	↓KEAP1, ↓MDA, ↑CAT, ↑GPx, ↑SOD, ↑GSH, ↑GST	Ren et al. (2019)
Mouse (BALB/c)	Obesity induced by high fat diet	50 mg/kg (24 days)	—	Methylcellulose	Gavage	↓MDA, ↑CAT, ↑GSH, ↑SOD	Khatun et al. (2023)
	Ethanol induced by hepatotoxicity	40 and 50 mg/kg (5 days)	—	Maltose solution	Gavage	↓gp91^phox^, ↓NOX, ↓p47^phox^, ↓ROS production, ↑CAT, ↑GPx, ↑GSH, ↑GSSG, ↑NRF2	Yan et al. (2016)
Mouse (C76/BL6)	Femoral artery I/R	100 and 200 mg/kg (2 days)	—	Saline	Intraperitoneal injection	↑NRF2, ↑GPX4, ↑SLC7A11	Wang, F. et al. (2023)
Rat (SD) hippocampal neuronal cell*	Cerebral ischemia induced by OGD/R	0.1, 1, 10, and 20 µM (24 h)	Nimodipine	—	—	↓ROS production, ↑SOD, ↓MDA	Cao et al. (2016)
Human*	Acute myeloid leukemia	10 µM (1 h)	—	—	—	↓CAT, ↑GPx, ↓MDA, ↓SOD, ↓TOS	Haddadi et al. (2023)

Notes: The table organizes *in vivo* and *ex vivo* experiments, where upward and downward arrows respectively indicate upregulation and downregulation of the target by SA. Experiments marked with an asterisk are *ex vivo*.

Abbreviations: 4-HNE, 4-hydroxynonenal; CAT, catalase; GPx, glutathione peroxidase; GSH, glutathione; GST, glutathione-S-transferase; H/R, hypoxia/reoxygenation; iNOS, inducible nitric oxide synthase; I/R, ischemia/reperfusion; L-NAME, N^ω^-nitro-L-arginine methyl ester; MDA, malonaldehyde; NDMA, N-nitrosodimethylamine; NQO1, NADPH, quinone acceptor oxidoreductase 1; NOX, NADPH, oxidase; NRF2, nuclear transcription factor-erythroid 2 related factor; OGD/R, oxygen-glucose deprivation/reoxygenation; ROS, reactive oxygen species; SLC7A11, solute carrier family 7 member 11; SOD, superoxide dismutase; TAS, total antioxidant status; TOS, total oxidant status.

### 3.1 Structure-activity relationship of SA

In both *in vivo* and *in vitro* settings, the radical scavenging activity of a metabolite is largely determined by its specific molecular structure (Nimse and Pal, 2015). The position and number of hydroxyl groups play a crucial role in enhancing the antioxidant capacity of phenolic acids (Eom et al., 2012).

Gallic acid (GA), characterized by a trihydroxybenzene ring with hydroxyl at positions 3, 4, and 5, is converted to syringic acid (SA) through methylation of the hydroxyl groups at positions 3 and 5, forming methoxy groups (Figure 1). Although the substitution of methoxy groups reduces the antioxidant capacity-evidenced by studies showing GA has a stronger antioxidant capacity than SA (Chen, 2011; Skroza et al., 2022), the presence of methoxy groups in SA still contributes to its significant antioxidant capability. Methoxy groups act as electron-donating substituents that stabilize the phenoxy radical formed during free radical scavenging (Razzaghi-Asl et al., 2013). Specifically, the methoxy groups at positions 3 and 5 (-OCH_3_) increase the bond dissociation enthalpy (BDE) of the O–H bond in the phenolic moiety, promoting the stabilization of the phenoxy radical generated upon reaction with free radicals (Chen et al., 2020; Zhang et al., 2002). Notably, the presence of two methoxy groups flanking the phenoxy radical enhances its stability compared to a single methoxy substituent (Bors et al., 2002). Additionally, the radical-scavenging ability of phenolic metabolites is linked to their acidity, which facilitates proton donation, and the delocalized π-electrons of their benzene ring, which enables electron transfer while maintaining structural stability (Parmar et al., 2011). This dual mechanism—proton donation and electron transfer—underscores the intricate balance that governs SA’s antioxidant efficacy.

### 3.2 SA modulates the generation of free radicals

Free radicals are highly active intermediates characterized by the presence at least one unpaired electron in their outer shell. Excessive free radicals production within the body acts a catalyst for damage to biological molecules, including DNA, proteins, and cellular membranes, thereby contributing to the development of various diseases (Liu, 2020; Phaniendra et al., 2015). In biological systems, free radicals primarily originate from ROS and RNS (Jomova et al., 2023). Common ROS include hydroxyl radicals (·OH), singlet oxygen (^1^O_2_), hydrogen peroxide (H_2_O_2_), and superoxide anion (O_2_^.−^). NADPH oxidases (NOXs), a family of membrane-bound enzymes present in various cell types, catalyze the transfer of electrons from NADPH to oxygen molecules, generating ROS, primarily O_2_^.−^ and the subsequently produced H_2_O_2_ (Bedard and Krause, 2007). SA reduces ROS formation by downregulating the expression of p47^phox^ and gp91^phox^ induced by alcohol in the liver. These two components, the cytosolic and membrane components of NOX, respectively, contribute to decreasing the formation of ROS (Yan et al., 2016). In the broader context of oxidative stress and inflammation, lipoxygenases (LOXs) play a pivotal role by catalyzing the metabolism of arachidonic acid to produce eicosanoids, thereby contributing to the ROS pool (Kattoor et al., 2017; Wang et al., 2020). SA acts as an effective LOX inhibitor, exhibiting an IC_50_ value of 0.009 mM (Habza-Kowalska et al., 2021).

Common RNS include nitric oxide (NO) and peroxynitrite (ONOO^−^). NO is primarily produced by NO synthase (NOS), which exists in three isoforms: endothelial NOS (eNOS), inducible NOS (iNOS), and neuronal NOS (nNOS) (Stuehr and Haque, 2019). The interaction of O_2_^.−^ with NO yields ONOO^−^, a highly reactive oxidant capable of inducing oxidative and nitrative damage (Matsubara et al., 2015). SA reduces NO production by downregulating iNOS expression (Güzelad et al., 2021; Khatun et al., 2023).

### 3.3 SA modulates the endogenous antioxidant defense system

The redox equilibrium of cells is maintained by a complex endogenous antioxidant defense mechanism, comprising enzymatic antioxidants like SOD, catalase (CAT), and glutathione peroxidase (GPx), as well as non-enzymatic components such as glutathione (GSH), and iron-binding proteins such as ferritin and transferrin (Poljsak et al., 2013). SOD plays a critical role by catalyzing the dismutation of superoxide radical anion, a harmful by-product of cellular metabolism, into H_2_O_2_, which is less reactive (Wang et al., 2018). CAT then rapidly decomposes H_2_O_2_ into water and molecular oxygen preventing its accumulation and potential conversion into more reactive species (Nandi et al., 2019). GPx, a selenium-containing enzyme, further detoxifies H_2_O_2_ and organic hydroperoxides by reducing them to water or their corresponding alcohols, utilizing GSH as a cofactor (Brigelius-Flohé and Flohé, 2020). GSH, a tripeptide composed of glutamate, cysteine, and glycine, acts as a critical antioxidant by directly scavenging free radicals and reactive intermediates. It also serves as a substrate for GPx-catalyzed reactions, where it is oxidized to glutathione disulfide (GSSG) during the reduction of H_2_O_2_, thereby mitigating ROS-induced damage and maintaining cellular redox homeostasis (Forman et al., 2009).

Oxidative stress is commonly defined as an imbalance between the production and elimination of ROS. The imbalance not only increases free radicals but also impairs antioxidant defense system, leading to reduced activity of these enzymes and molecules (Pérez-Torres et al., 2021). Consequently, these antioxidant indicators are often used to assess the efficacy of antioxidants. Studies have shown that SA significantly enhances the activity of these indicators in response to oxidative stress damage across various systems, including cardiovascular (Güven et al., 2015; Kumar et al., 2012), neural (Cao et al., 2016; Mallan and Singh, 2023), endocrine (Sherkhane et al., 2023), and reproductive systems (Akarsu et al., 2023; Demir, 2023).

Within the cancer microenvironment, although oxidants play roles through various stages of tumor development, excessive accumulation of cellular ROS leads to oxidative stress, which can trigger apoptosis. This mechanism not only limits metastasis but also prevents tumorigenesis (Forman and Zhang, 2021). Furthermore, the surplus of ROS damages cellular components such as DNA, proteins, lipids, membranes, and organelles, acting as a pivotal signal to activate the apoptotic pathways. Increasing evidence indicates that inducing the accumulation of intracellular ROS is a primary strategy underlying the cytotoxicity of anticancer drugs (Redza-Dutordoir and Averill-Bates, 2016). Phenolic metabolites are believed to exert their anticancer activities through such mechanisms (Yang et al., 2020). For instance, high doses of GA in cell culture media can convert O_2_ into H_2_O_2_, increasing ROS levels and inducing selective cytotoxicity in cancer cells (Wang et al., 2016). Both *in vivo* and *in vitro* studies, Yang et al. demonstrated that SA can enhance the production of ROS in tumor cells of rat lung cancer, leading to apoptosis (Yang et al., 2020). Similarly, Pei et al. reported that SA reduces the activity of antioxidant enzymes in gastric cancer cells, induces OS, and selectively promotes apoptosis, potentially through the depolarization of mitochondrial membrane potential (Pei et al., 2021). Additionally, oral administration of SA to rats with colorectal cancer increased ROS production, decreased antioxidant enzyme activity, and caused DNA damage, as evidenced by elevated levels of 8-OHdG and AP-sites, both of which are significant indicators of oxidative DNA damage (Mihanfar et al., 2021).

In summary, SA exhibits a bidirectional modulation of antioxidant enzyme activity. Specifically, in the context of cancer, it promotes oxidative stress, modulates antioxidant enzyme activity, and induces apoptosis, consistent with the general activities of polyphenols (Chimento et al., 2023). However, the factors determining the selective pro-/anti-oxidant activity of SA in various disease contexts remain unclear. Current research on SA’s anticancer activities has primarily focused on apoptosis. The underlying mechanisms contributing to this duality will be explored in section “5.2 apoptosis”.

### 3.4 SA modulates KEAP1/NRF2/HO-1 signaling pathway

**FIGURE 2:**
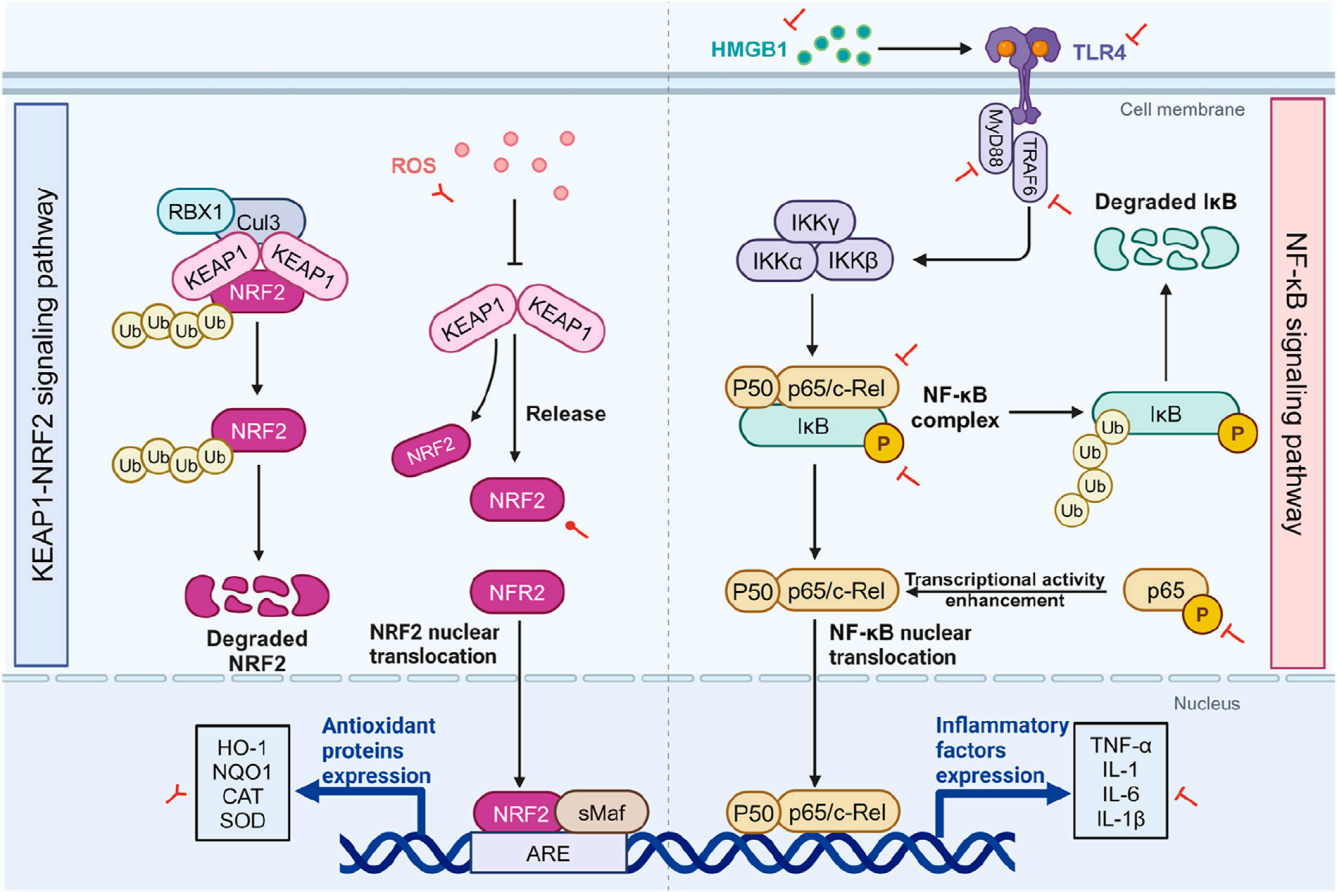
The impact of SA on the KEAP1/NRF2 and NF-κB pathways. The two sides of the diagram respectively depict the KEAP1/NRF2 pathway (left) and the NF-κB pathway (right), as well as the effects of SA on them. Under normal conditions, NRF2 is kept at low levels through its binding with KEAP1 in the cytoplasm, leading to its ubiquitination and degradation. However, OS alters KEAP1, enabling NRF2 accumulation and nuclear migration, where it enhances antioxidant gene expression including HO-1, NQO1, CAT, and SOD. Within the NF-κB signaling cascade, cellular stressors such as the interaction of TLR4 with extracellularly released HMGB1 initiate a complex series of events. This cascade involves the recruitment of the adaptor protein MyD88 and TRAF6, which in turn activates the IKK complex. The activation of IKK results in the targeted phosphorylation of IκB proteins, tagging them for proteasomal degradation and thereby liberating the NF-κB p50/p65 dimers. Under OS and inflammation-related diseases, SA inhibits ROS and activates NRF2, enhancing antioxidant gene expression while concurrently attenuating the NF-κB pathway by reducing the expression of p-IκB-α, p65 and p-p65, as well as downregulating upstream signaling components such as TLR4, HMGB1, MyD88, and TRAF6. In the context of cancer, SA leads to an increase in ROS and decreases the expression of antioxidant genes. Red circular, T-shaped, and bifurcated arrows represent promotion, inhibition, and bidirectional effect by SA, respectively. ARE, antioxidant response element; CAT, catalase; Cul3, cullin-3; HMGB1, high mobility group box 1; HO-1, heme oxygenase-1; IKK, IκB kinase; IκB, inhibitor κB; KEAP1, cytoplasmic kelch-like epichlorohydrin-associated protein 1; IL, interleukin; MyD88, myeloid differentiation primary response 88; NF-κB, Nuclear factor-kappa B; NQO1, NADPH quinone oxidoreductase enzyme; NRF2, nuclear factor erythroid 2-related factor 2; RBX1, ring-box 1; ROS, reactive oxygen species; sMAF, small musculoaponeurotic fibrosarcoma; SOD, superoxide dismutase; TLR, toll-like receptor; TNF-α, tumor necrosis factor-α; TRAF6, tumor necrosis factor receptor-associated factor 6. Created in BioRender. Zhejun, Z. (2024) BioRender.com/q25k994.

The Kelch-like ECH-associated protein 1 (KEAP1)/nuclear factor-erythroid related factor 2 (NRF2) signaling pathway is a key regulator of the antioxidant defense system, playing a critical role in protecting cells from oxidative stress. It implicated in various inflammatory diseases, including cancer, neurodegenerative diseases, cardiovascular diseases, and aging (Fão et al., 2019; Lu et al., 2016; Tu et al., 2019). NRF2 is a transcription factor that activates the expression of a range of antioxidant genes, reducing ROS levels and mitigating oxidative stress damage (Hybertson et al., 2011). Under normal condition, NRF2 binds with KEAP1 in the cytoplasm. As part of the E3 ubiquitin ligase complex that includes Cullin 3 (Cul3) and RING-box protein 1 (RBX1), KEAP1 promotes the ubiquitination and subsequent proteasomal degradation of NRF2, thus maintaining NRF2 low levels. However, during oxidative stress or ROS exposure, these oxidants modify KEAP1’s structure, impairing its ability to capture NRF2. Consequently, NRF2 escapes degradation, accumulates, and translocates to the nucleus. In the nucleus, NRF2 forms heterodimers with small musculoaponeurotic fibrosarcoma (sMaf) proteins and binds to antioxidant response elements (ARE), activating the expression of antioxidant genes, including heme oxygenase-1 (HO-1) and NAD(P)H quinone dehydrogenase 1 (NQO1) (Figure 2) (Tonelli et al., 2018).

In an early study by Yan et al., SA intake significantly increased the expression of NRF2 in liver cell nuclei in a mouse model of ethanol-induced hepatotoxicity. This suggests that SA may stabilize intracellular NRF2 molecules, promote its translocation into the nucleus, and regulate antioxidant-related genes (Yan et al., 2016). Similarly, SA ingestion in rats with pulmonary fibrosis significantly increased NRF2 protein expression in lung tissue (Somade et al., 2022). Comparable results were observed in models of testicular damage (Akarsu et al., 2023) and diabetic nephropathy (Sherkhane et al., 2023), where SA enhanced activity of NQO1, a downstream target of NRF2. Furthermore, Ekhtiar et al. found that a higher dosage of SA (50 mg/kg) significantly upregulated the expression of the antioxidant genes NRF2, HO-1, and NQO1, mitigating oxidative and inflammation in a model of experimental ulcerative colitis, while a lower dosages (10 mg/kg) did not elicit the same effect, indicating that SA dose-dependent activation of NRF2 pathway (Ekhtiar et al., 2023).

HO-1, regulated by NRF2, catalyze the rate-limiting step in heme breakdown, producing ferrous ions, biliverdin, and carbon monoxide (Loboda et al., 2016). Biliverdin is rapidly converted into bilirubin by biliverdin reductase (BVR), both of which are widely recognized for their anti-inflammatory and antioxidant properties (Vijayan et al., 2018). Furthermore, iron homeostasis mechanisms, including the increased in ferritin synthesis, facilitate the safe storage and utilization of these released ferrous ions, protecting cells from oxidative damage (Sha et al., 2023). This aspect will be further discussed in section “5.3 ferroptosis”. Besides regulating antioxidant enzyme expression through NRF2 activation, HO-1upregulation can also enhance the expression of other antioxidant enzymes, including SOD, GPx, and CAT (Ryter, 2022). Studies have shown that HO-1 knockout animal models exhibit elevated lipid peroxidation and oxidized protein levels (Mansouri et al., 2022), highlighting HO-1’s critical role in combating OS and inflammation. Recent *in vivo* studies have demonstrated that SA activates the NRF2/HO-1 signaling pathway in inflammatory models. Ekhtiar et al. reported that ulcerative colitis increased pro-inflammatory cytokines TNF-α and IL-1β, as well as decreased expression of the antioxidant genes HO-1, NRF2, and NQO1. Treatment with SA was able to reverse these effects in a dose-dependent manner (Ekhtiar et al., 2023). Additionally, SA improves ovarian toxicity in rats exposed to CIS, balancing the levels of the antioxidant system, potentially through NRF2/HO-1 pathway activation (Demir, 2023). In summary, SA’s structure underlies its antioxidant capacity, reducing free radical production, enhancing the endogenous antioxidant defense system, and activating the KEAP1/NRF2/HO-1 pathway (Figure 2).

## 4 Anti-inflammatory mechanisms of SA

Inflammation is the body’s innate response to infection or injury and can be categorized into two types: acute and chronic. Acute inflammation activates the immune system to facilitate rapid recovery clearing invaders and repairing tissues, which is generally beneficial to the host (Joffre and Hellman, 2021; Reuter et al., 2010). However, unresolved inflammation can progress to chronic inflammation, which is associated with various diseases, including cardiovascular diseases (Senoner and Dichtl, 2019), neurodegenerative diseases (Teleanu et al., 2022), diabetes (Poznyak et al., 2020), and cancer (Caliri et al., 2021).

Various inflammatory stimuli trigger the migration of immune cells, such as macrophages and neutrophils, to the site of injury, where they release inflammatory mediators including NO, prostaglandin E2 (PGE2), tumor necrosis factor-alpha (TNF-α), and interleukin-1 beta (IL-1β) (Medzhitov, 2008). This process promotes tissue repair and antigen clearance but may also lead to pain and edema. Prolonged inflammation leads to elevated levels of pro-inflammatory mediators such as iNOS, cyclooxygenase-2 (COX-2), PGE2, and various cytokines such as TNF-α, IL-1β, and IL-6, is observed (Alessandri et al., 2013; Watson et al., 2017). Furthermore, the activation of T cells fosters the production of lymphokines, including IL-2 and interferon-γ (IFN-γ), further mobilizing immune cells to combat pathogens and repair tissue (Ding et al., 2022). The specific anti-inflammatory mechanisms of SA will be discussed next, focusing on its regulation of inflammatory mediators and molecular signaling pathways. The effects of SA on inflammation-related targets are summarized in Table 2.

**TABLE 2:** The effects of SA on inflammation-related targets in different types of experiments.

Species and strain	Model or disease	Tested dosage (duration)	Positive control	Negative control	Administration approach	Target	References
Rat (Sprague-Dawley)	Ovarian injury induced by cisplatin	5 and 10 mg/kg (3 days)	—	DMSO	Intraperitoneal injection	↓HMGB1, ↓MPO, ↓NF-κB p65, ↓TNF-α	Demir (2023)
	Testicular injury induced by I/R	50 and 100 mg/kg (2.5 h)	—	DMSO	Intraperitoneal injection	↓HMGB1, ↓IL-6, ↓MPO, ↓NF-κB p65, ↓TNF-α	Demir et al. (2023)
Rat (Wistar)	Hepatic encephalopathy induced by thioacetamide	50 and 100 mg/kg (14 days)	—	—	Gavage	↑IL-10, ↓IL-1β, ↓iNOS, ↓NF-κB, ↓TNF-α	Ferah Okkay et al. (2022)
	Ulcerative colitis induced by acetic acid	10, 25, and 50 mg/kg (5 days)	Dexamethasone	—	Gavage	↓IL-1β, ↓IL-6, ↓iNOS, ↓NF-κB, ↓TLR4	Ghasemi-Dehnoo et al. (2024)
	Ovarian damage induced by cyclophosphamide	5, 10, and 20 mg/kg (14 w)	—	Saline	Intraperitoneal injection	↓COX-2, ↓IL-1β, ↓IL-6, ↓iNOS, ↓NF-κB, ↓PGE2, ↓TNF-α	Liu, J. et al. (2021)
	Autism induced by valproic acid	25, 50, and 100 mg/kg (28 days)	—	Saline	Intraperitoneal injection	↓IL-6, ↓TNF-α	Mallan and Singh (2023)
	Diabetic rats induced by streptozotocin	2.5% and 5% (14 days)	—	—	Topical application	↑CD31+, ↑CD68+, ↑IL-10, ↓IL-2, ↓IL-8, ↓IL-1β, ↓NF-κB p65, ↓TNF-α	Ren et al. (2019)
	Cardiotoxicity induced by isoproterenol	50 mg/kg SA and 25 mg/kg SA + resveratrol (30 days)	Gallic acid	DMSO	Gavage	↓NF-κB, ↓TNF-α	Manjunatha et al. (2020)
	Lung fibrotic induced by NDMA	50 mg/kg (28 days)	Ascorbic acid	Saline	Gavage	↓AKT, ↓IL-1β, ↓NF-κB, ↓PI3K, ↓TNF-α	Somade et al. (2022)
Mouse (BALB/c)	Colitis induced by DSS	25 mg/kg (7 days)	—	—	Gavage	↓CD68+, ↓COX-2, ↓IL-1β, ↓IL-6, ↓iNOS, ↓MPO, ↓NF-κB p65, ↓p-IκB-α, ↓TNF-α	Fang et al. (2019)
	Liver injury induced by concanavalin A	0.1, 1, 10 mg/kg (24 h)	—	—	Intraperitoneal injection	↓IFN-γ, ↓IL-6, ↓TNF-α	Itoh et al. (2009)
	Ethanol induced by hepatotoxicity	40 and 50 mg/kg (5 days)	—	Maltose solution	Gavage	↓COX-2, ↓iNOS, ↓IL-6, ↓NF-κB p65, ↓TNF-α	Yan et al. (2016)
Mouse (C57BL/6 J)	Obesity induced by high fat diet	0.05% SA 0.5 g/kg HFD diet (16 w)	—	Normal diet	Diet	↓IFN-γ, ↓IL-6, ↓MyD88, ↓NF-κB, ↓TLR4, ↓TNF-α	Ham et al. (2016)
	Colitis induced by dextran sulfate sodium	50 mg/kg (7 days)	Fecal microbiota transplantation	PBS	Gavage	↓IL-6, ↓IL-10, ↓IL-17A, ↓TNF-α, ↓TRAF6	Luo et al. (2023)
Mouse hippocampal and cerebrocortical slices (Swiss) *	Depression induced by glutamatergic excitotoxicity	1 mg/kg (7 days)	Fluoxetine	Distilled water	Gavage	↑p-GSK-3β, ↓p-AKT (Ser473)	Dalmagro et al. (2019)
Mouse chondrocytes (C57BL/6) *	Osteoarthritic cartilage degradation	1, 5, 10, and 50 μM (1 and 2 days)	—	—	—	↓p-IκB-α, ↓IL-1β, ↓p-p65, ↓TNF-α	Wang, M. et al. (2023)

Notes: The table organizes *in vivo* and *ex vivo* experiments, where upward and downward arrows respectively indicate upregulation and downregulation of the target by SA. Experiments marked with an asterisk are *ex vivo*.

Abbreviations: AKT, protein kinase B; COX-2, cyclooxygenase-2; DSS, dextran sulfate sodium; GSK-3β, glycogen synthase kinase 3β; HFD, high-fat and high-cholesterol diet; HMGB1, high mobility group box 1; IFN-γ, interferon-γ; IL, interleukin; iNOS, inducible nitric oxide synthase; MPO, myeloperoxidase; MyD88, myeloid differentiation primary response 88; NDMA, N-nitrosodimethylamine; NF-κB, nuclear factor kappa B; PGE2, prostaglandin E2; p-IκBα, phosphorylated-inhibitor of kappa B alpha; TLR4, toll-like receptor 4; TNF-α, tumor necrosis factor-alpha; TRAF 6, TNF receptor associated factor 6.

### 4.1 SA modulates inflammatory mediators

Cytokines are key regulators of the immune system, comprising both pro-inflammatory and anti-inflammatory types, which play crucial roles in intercellular communication, maintaining homeostasis, and disease progression (Opal and DePalo, 2000). Pro-inflammatory cytokines, such as TNF-α, IL-6, IL-1β, and IFN-γ, promote inflammatory responses and the development of chronic diseases. Conversely, anti-inflammatory cytokines like IL-10 and IL-4 inhibit the inflammatory process by regulating the responses of pro-inflammatory cytokines, maintaining the balance of the immune system (Henshaw et al., 2021; Propper and Balkwill, 2022). The regulation of balance of these cytokines is essential for controlling inflammatory responses. Numerous studies have shown that in various animal models, interventions with SA can exert anti-inflammatory activities by simultaneously downregulating pro-inflammatory cytokines including TNF-α, IL-6, IL-1β, IFN-γ and upregulating anti-inflammatory factors such as IL-10.

In addition to cytokines, other key molecules like COX-2 and PGE2 also play crucial roles in the regulation of inflammatory responses. COX-2, an enzyme upregulated during inflammation, catalyzes the production of prostaglandins, especially PGE2, which is an important mediator of typical symptoms such as inflammation, pain, and fever (Kawahara et al., 2015). In *in vivo* inflammation models, such as drug-induced liver damage, ovarian injury, and colitis, SA intake significantly downregulated the activity of COX-2 and PGE2 (Fang et al., 2019; Gheena et al., 2022; Liu, J. et al., 2021).

Myeloperoxidase (MPO), an enzyme abundantly present in neutrophils, catalyzes the production of potent oxidants such as hypochlorous acid, which contributes to ROS generation. Activated neutrophils utilize ROS produced by MPO to further modulate the release of inflammatory cytokines, exacerbating the inflammatory response (Aratani, 2018; Hawkins, 2020). In two reproductive system studies by Demir et al., regardless of whether it was a rat ovarian damage model or a testicular damage model, SA intake significantly reduced the activity of MPO (Demir, 2023; Demir et al., 2023). Additionally, the increased activation of immune cells, including neutrophils, macrophages, and T lymphocytes, enhances their recruitment and activation at inflammation site, leading to elevated pro-inflammatory cytokines production (Ozga et al., 2021). Fang et al. demonstrated that in a dextran sulfate sodium-induced colitis mouse model, SA effectively regulated both the activity and expression of MPO and CD68^+^, a macrophage marker, suggesting suppression of immune cell activation (Fang et al., 2019). Interestingly, a study on wound healing in diabetic rats produced opposite results, where the topical application of SA significantly upregulated the protein expression of CD68 and the endothelial tissue marker CD31, promoting wound healing (Ren et al., 2019). This discrepancy may be explained by the fact that cytokines produced by macrophages are essential for fibroblast proliferation and extracellular matrix deposition; their ablation can impair healing and hinder functional recovery (Kessenbrock et al., 2010).

Collectively, these studies suggest that the effects of SA on inflammation are context-dependent. In inflammatory disease models, SA’s anti-inflammatory properties help alleviate tissue damage and inflammation. Conversely, in tissue repair and regeneration contexts, SA promotes wound healing by activating immune cells and enhancing angiogenesis.

### 4.2 SA modulates NF-κB pathway

Nuclear factor kappa B (NF-κB) is a key signaling pathway that regulates inflammatory responses, immune reactions, and cell survival (Mitchell et al., 2016; Zinatizadeh et al., 2021). The NF-κB family consists of five protein monomers: p65 (RelA), RelB, cRel, p50, and p52, which form homodimers or heterodimers that regulate gene expression by specifically binding to DNA. The classical activation pathway of NF-κB is triggered by upstream signals, including IL-1, TNF, and TLR4 stimulation, which activate the IκB kinase (IKK) complex. IKKβ phosphorylates IκB protein, leading to its degradation and the release of NF-κB dimers, typically the p50/p65 dimer (Zinatizadeh et al., 2021). The NF-κB dimer then enters the nucleus, binds to specific sequences of DNA, recruits coactivators, and initiates the transcription of target genes (Figure 2).

Currently, existing *in vivo* experiments have widely demonstrated that SA downregulates the expression of NF-κB. For example, in an ethanol-induced liver toxicity mouse model, SA administration reduced NF-κB p65 expression in a dose-dependent manner, without significant impact observed on p50 (Yan et al., 2016). This reduction has also been noted across several inflammatory models, including reproductive system injuries (Demir, 2023; Demir et al., 2023), diabetes (Ren et al., 2019), and colitis (Fang et al., 2019). p65 can be further phosphorylated to p-p65, enhancing its transcriptional activity (Xu, X. et al., 2021). SA can also reduce the phosphorylation of p65 (Wang, M. et al., 2023; Xiang and Xiao, 2022). Meanwhile, once IκB-α is phosphorylated, it is rapidly degraded via the proteasomal pathway, releasing the NF-κB dimer bound to it. Furthermore, SA downregulates p-IκB-α expression in osteoarthritis and colitis models, preventing NF-κB activation (Fang et al., 2019; Wang, M. et al., 2023).

The activation of NF-κB also depends on the recognition and transmission of upstream signals. TLR4, a pattern recognition receptor on the cell surface, recognizes pathogen-associated molecular patterns like LPS, triggering downstream signaling and activating the IKK complex (Ciesielska et al., 2021). Similarly, high mobility group box 1 (HMGB1), an intracellular DNA-binding protein, can be released extracellularly upon cell stress or death, interacting with TLR4 to amplify inflammatory signaling (Chen et al., 2022). SA can also downregulate the expression of both HMGB1 (Demir, 2023; Demir et al., 2023) and TLR4 (Ghasemi-Dehnoo et al., 2024) in inflammation-related models. Additionally, myeloid differentiation primary response 88 (MyD88) and tumor necrosis factor receptor-associated factor 6 (TRAF6) are pivotal in bridging TLR4 activation with NF-κB-mediated cellular responses (Cohen and Strickson, 2017). Upon TLR4’s recognition of its ligand, MyD88 is swiftly recruited to the receptor complex, followed by TRAF6, and together they transmit the activation signal to downstream signaling molecules (Deguine and Barton, 2014). Ham et al. also evaluated the expression of TLR4 and MyD88 in the liver tissues of obese mice. SA significantly reduced the gene expression of both, suggesting that by downregulating the TLR4-MyD88 pathway, it decreases the gene expression of pro-inflammatory cytokines in the liver, including TNF-α and IL-6 (Ham et al., 2016). The inhibitory effect of SA on TRAF6 has also been observed in a colitis model (Luo et al., 2023).

In summary, SA downregulates NF-κB signaling by reducing NF-κB p65 expression and phosphorylation, lowering p-IκB-α levels, and inhibiting nuclear transcription. Additionally, it impacts upstream regulatory mechanisms by suppressing TLR4, HMGB1, and adaptor proteins like MyD88 and TRAF6, highlighting its role in modulating inflammatory responses (Figure 2).

## 5 Potential crosstalk among different pathways involving SA

Current evidence suggests that SA exhibits significant antioxidant and anti-inflammatory activities. However, its direct molecular targets remain unclear. While SA is well-known for its antioxidant properties, typical of phenolic acids, stronger evidence supports its anti-inflammatory effects. If SA’s antioxidant activity arise from its inherent ROS-scavenging ability, then the reduced oxidative stress should, in theory, restore KEAP1’s ability to capture NRF2. Studies often report increased NRF2 levels following SA intervention, suggesting that SA may stabilize NRF2. Notably, potential publication bias, particularly regarding NF-κB pathway research, cannot be ruled out. Therefore, we will examine NRF2’s upstream regulators and pathway crosstalk to identify SA’s potential direct targets.

### 5.1 The potential role of SA in modulating crosstalk between NRF2 and NF-κB

Oxidative stress and inflammation are two highly interconnected processes. NRF2 is a crucial molecule in regulating oxidative stress, while inflammation induced by oxidative stress is primarily triggered by the activation of NF-κB (Ahmed et al., 2017).

**FIGURE 3:**
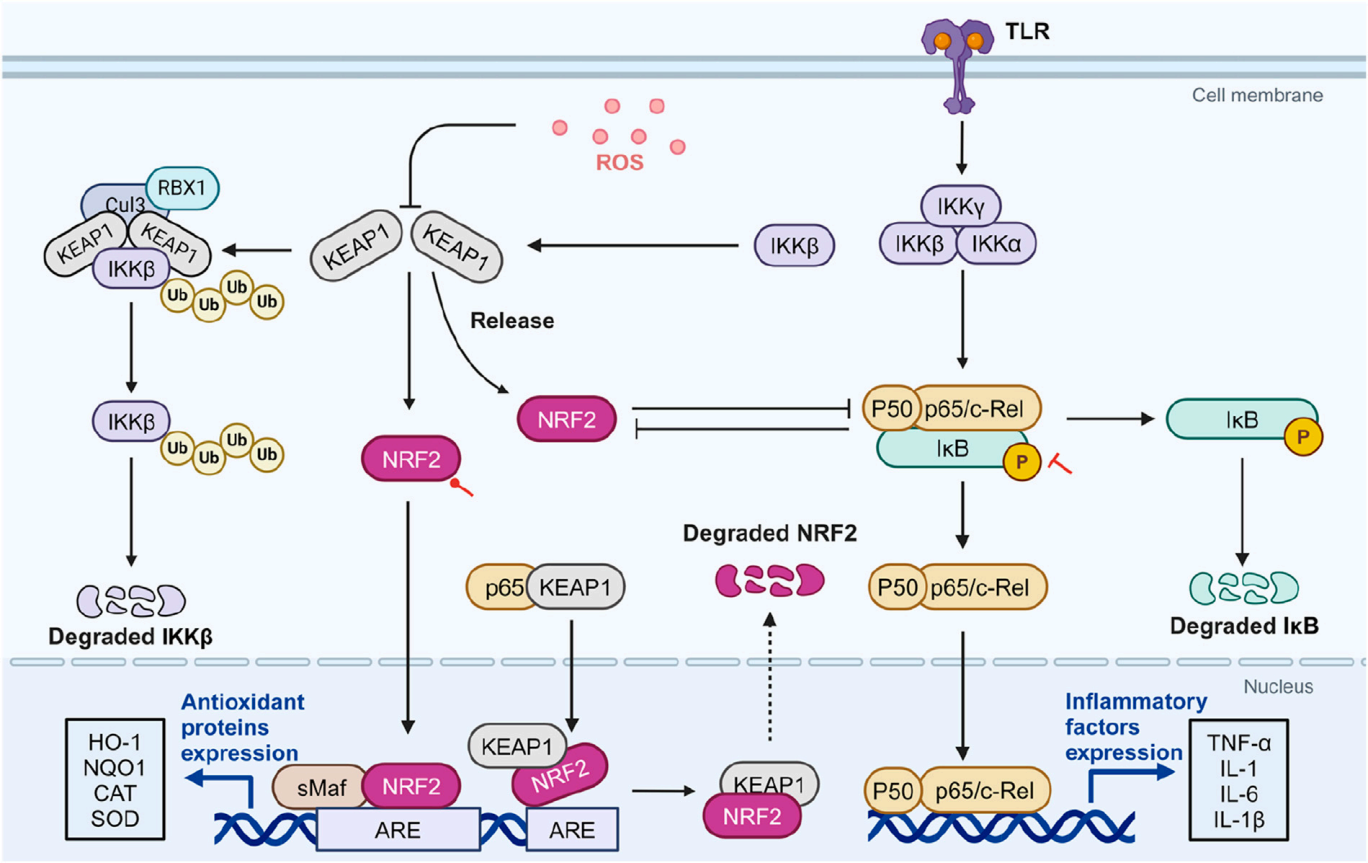
The potential role of SA in modulating crosstalk between NRF2 and NF-κB. ROS-rich conditions inhibit KEAP1, stabilizing IKKβ and leading to NF-κB activation through IκBα degradation. NRF2 inhibits NF-κB’s nuclear translocation by preventing IκBα degradation and reduces NF-κB activation by enhancing antioxidant defenses and lowering ROS levels. Conversely, NF-κB p65 can inhibit NRF2 activation by affecting ARE gene transcription and facilitating NRF2 degradation *via* interactions with KEAP1. In diseases related to OS and inflammation, SA activates NRF2 and inhibits NF-κB. Red circular and T-shaped arrows represent promotion and inhibition by SA, respectively. Dashed arrows indicate indirect effects. ARE, antioxidant response element; CAT, catalase; Cul3, cullin-3; HO-1, heme oxygenase-1; IKK, IκB kinase; IκB, inhibitor κB; KEAP1, cytoplasmic kelch-like epichlorohydrin-associated protein 1; IL, interleukin; NF-κB, Nuclear factor-kappa B; NQO1, NADPH quinone oxidoreductase enzyme; NRF2, nuclear factor erythroid 2-related factor 2; RBX1, ring-box 1; ROS, reactive oxygen species; sMAF, small musculoaponeurotic fibrosarcoma; SOD, superoxide dismutase; TLR, toll-like receptor; TNF-α, tumor necrosis factor-α. Created in BioRender. Zhejun, Z. (2023) BioRender.com/n67e000.

Their interaction is complex. Similar to NRF2, IKKβ can also bind to KEAP1, leading to its ubiquitination and proteasomal degradation. Under ROS-rich condition, KEAP1 is inhibited, stabilizing IKKβ, which phosphorylates and degrades I-κBα, thereby activating NF-κB (Lee et al., 2009). Conversely, the NRF2 pathway inhibits NF-κB nuclear translocation of by preventing the proteasomal degradation of IκB-α (Saha et al., 2020). On the other hand, NF-κB inhibits NRF2 activation by reducing the transcription of ARE genes (Cuadrado et al., 2018). NF-κB p65 additionally interacts with KEAP1, promoting its translocation to the nucleus. This process facilitates the separation of NRF2 from the ARE, leading to the increased ubiquitination and degradation of NRF2 (Kim and Jeon, 2022). This reciprocal negative regulation creates a complex interplay between NF-κB and NRF2 (Figure 3). SA’s potent antioxidant and anti-inflammatory activities may stem from a synergistic feedback loop involving NRF2 activation and NF-κB inhibition. Akarsu et al. concurrently evaluated the expression levels of these two molecules. In a rat model of testicular injury induced by lead acetate, a significant downregulation of NRF2 was observed alongside a significant upregulation of NF-κB, indicating the presence of oxidative stress and inflammation. SA intake was able to reverse this condition, suggesting that SA plays a role in regulating the NRF2/NF-κB signaling pathway (Akarsu et al., 2023). However, current research evaluating the simultaneous effects of SA intervention on both NRF2 and NF-κB remain limited. Given the intricate crosstalk between these pathways, the causal relationship is not fully understood, and both pathways may be influenced concurrently. Further studies are needed to elucidate the precise mechanisms and interactions involved.

### 5.2 The potential role of SA in modulating crosstalk among PI3K/AKT, NRF2, and NF-κB

NRF2 is an downstream targets of PI3K and can be phosphorylated by activated AKT (Yu and Xiao, 2021). One study indicated that under the action of PI3K inhibitors, there was a reduction in AKT1 phosphorylation, which in turn diminished the accumulation of carnosol-induced NRF2 protein (Martin et al., 2004). The PI3K/AKT/NRF2 pathway is considered a primary route for cells to counteract oxidative stress (Lai et al., 2020). Besides direct activation of NRF2, the PI3K/AKT pathway regulates the activity of NRF2 through glycogen synthase kinase 3β (GSK-3β), a widely distributed serine/threonine kinase (Grimes and Jope, 2001). Activated GSK-3β phosphorylates Fyn tyrosine kinase, promoting its nuclear translocation, where it phosphorylates NRF2 at Tyr568, facilitating its export *via* chromosomal region maintenance 1 (Crm1) and reducing its transcriptional activity (Culbreth and Aschner, 2018; Rojo et al., 2008). GSK-3β can also phosphorylate NRF2’s Neh6 domain, promoting KEAP1-independent degradation (Rada et al., 2011). Activated AKT phosphorylates GSK-3β at Ser9, leading to its inactivation (Zhou et al., 2013).

SA has been shown to activate the PI3K/AKT signaling pathway, reducing cellular ROS production and MDA content, thus protecting RGC-5 cells from oxidative damage induced by H_2_O_2_ (Song et al., 2016). In another oxidative injury model, the ischemia/reperfusion (I/R) injury rat model, intervention with SA significantly activated the PI3K/AKT/GSK-3β signaling pathway, thereby inhibiting mitochondria-induced apoptosis and alleviating myocardial damage (Liu, G. et al., 2020). In a glutamate-induced neurotoxicity model, SA can significantly increase the phosphorylation of PI3K, AKT, and GSK-3β, producing effects similar to fluoxetine. Moreover, inhibitors of PI3K and GSK-3β can negate these protective effects (Dalmagro et al., 2019). These findings suggest that SA acts as an activator of PI3K and GSK-3β, contributing to NRF2 elevation and antioxidant effect. However, whether SA directly interacts with PI3K and GSK-3β remains to be determined, as the specific binding sites are unknown.

However, in specific disease contexts, the effects of SA on the PI3K/AKT/NRF2 pathway are complex. Another downstream component of AKT is NF-κB (Yu et al., 2017). The PI3K/AKT pathway activates NF-κB by phosphorylating IKK, leading to the degradation of IκB (Burow et al., 2000; Yang et al., 2019). Somade et al. assessed the expression levels of the PI3K/AKT pathway, NRF2, and NF-κB in NDMA-induced pulmonary fibrosis (Somade et al., 2022). In this context, the PI3K/AKT pathway is activated, promoting both fibroblast proliferation and differentiation into myofibroblasts, which play an essential role in fibrotic disease (Conte et al., 2011; Fagone et al., 2011). In this model, the PI3K/AKT pathway is activated, NF-κB significantly increases with NRF2 downregulated. These changes are significantly reversed under SA intervention (Somade et al., 2022). Notably, despite PI3K/AKT activation typically upregulating NRF2, SA’s inhibition of PI3K/AKT coincided with NRF2 stabilization, suggesting that SA may directly stabilize NRF2 or that other upstream factors are involved.

**FIGURE 4:**
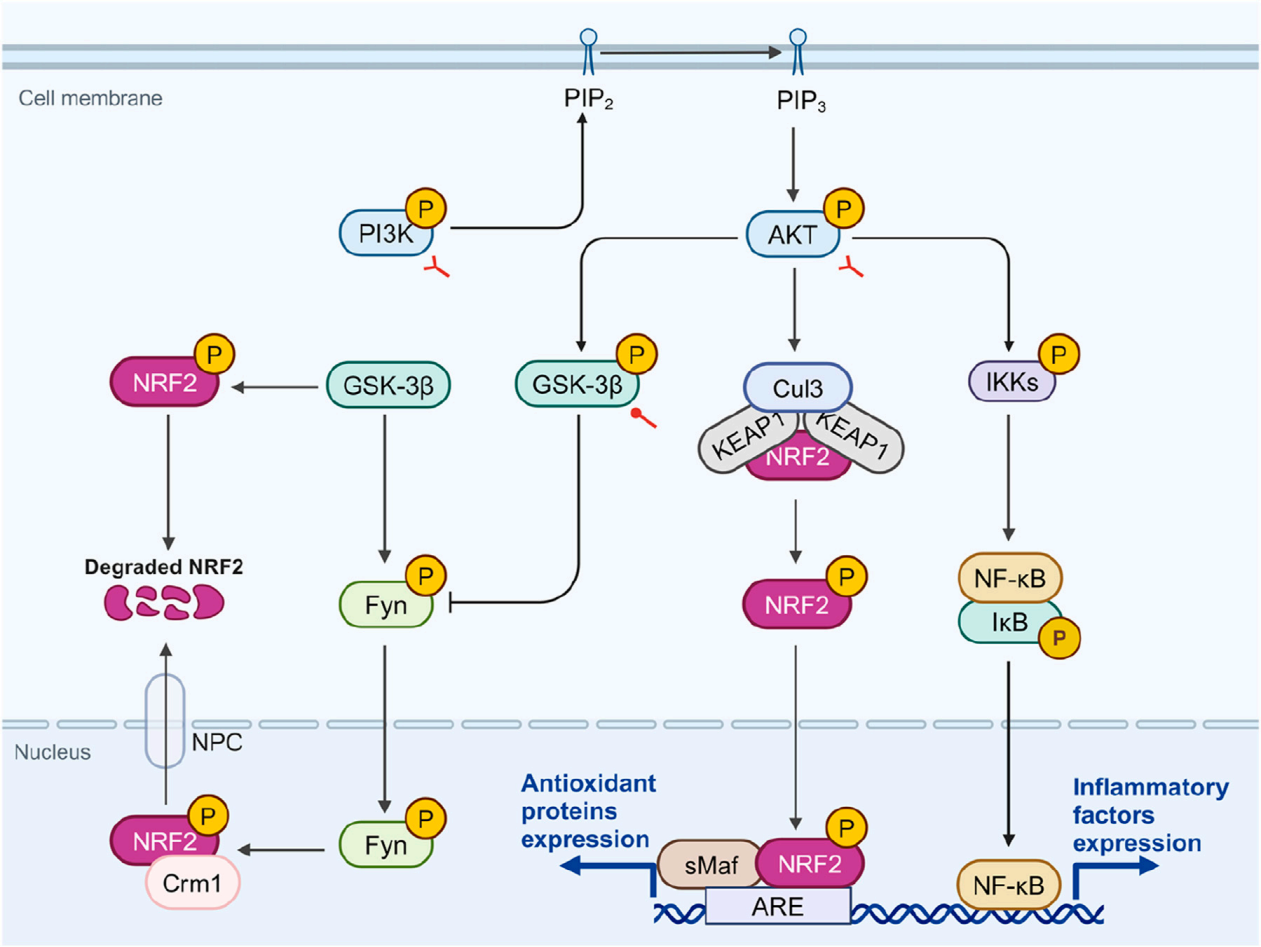
The potential role of SA in modulating crosstalk among PI3K, NRF2, and NF-κB. AKT phosphorylation, stimulated by PI3K, leads to the phosphorylation and inactivation of GSK-3β, which facilitates NRF2’s nuclear retention and antioxidant activity. GSK-3β can phosphorylate Fyn, leading to its nuclear translocation and subsequent phosphorylation of NRF2, which when associated with Crm1, diminishes NRF2’s transcriptional activity. Additionally, AKT-mediated phosphorylation of IKK promotes NF-κB activation and inflammatory response. In diseases related to OS and inflammation, SA promotes the phosphorylation of PI3K, AKT, and GSK-3β. However, in fibrotic diseases, SA inhibits the phosphorylation of PI3K and AKT. Red circular and bifurcated arrows represent promotion and bidirectional effect by SA, respectively. AKT, protein kinase B; ARE, antioxidant response element; Crm1, chromosomal region maintenance 1; Cul3, cullin-3; Fyn, fyn tyrosine kinase; GSK-3β, glycogen synthase kinase 3β; IKK, IκB kinase; IκB, inhibitor κB; NF-κB, Nuclear factor-kappa B; NPC, nuclear pore complex; NRF2, nuclear factor erythroid 2-related factor 2; PI3K, phosphatidylinositol-4,5-bisphosphate 3-kinase; PIP2, phosphatidylinositol 4,5-bisphosphate; PIP3, phosphatidylinositol 3,4,5-trisphosphate; sMAF, small musculoaponeurotic fibrosarcoma. Created in BioRender. Zhejun, Z. (2023) BioRender.com/r78r058.

Overall, SA appears to stabilize NRF2 directly while also acting as an activator of PI3K and GSK-3β (Figure 4). However, its effects may vary depending on disease contexts. The causal relationship between SA’s anti-inflammatory and antioxidant effects remains unclear, necessitating further research to identify its direct molecular targets.

## 6 The regulatory role of SA in OS and inflammation-related cellular pathological processes

**FIGURE 5:**
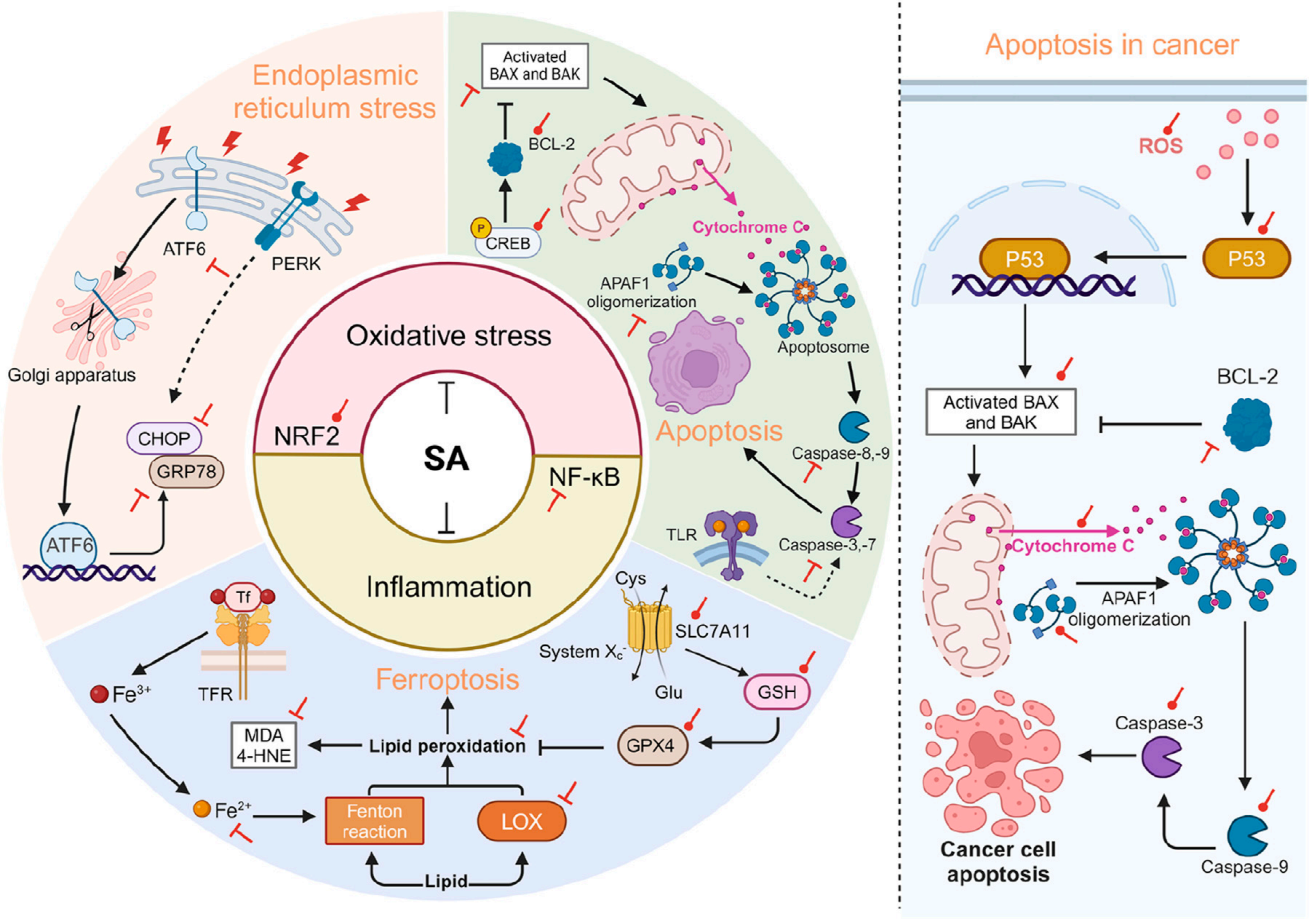
SA is involved in regulating cellular pathological processes related to OS and inflammation. The left side of the diagram illustrates that SA primarily acts to mitigate cellular pathological processes through its antioxidant and anti-inflammatory effects, achieved by enhancing NRF2 and suppressing NF-κB. Specifically, SA alleviates ERS by suppressing the expression of ATF6, CHOP, and GRP78. In terms of apoptosis, SA exerts its anti-apoptotic effect through the mitochondrial-dependent pathway, which includes upregulating Bcl-2 and downregulating Bax, Caspase-3, 8, and APAF1. SA also promotes the phosphorylation of CREB, an upstream activator of BCL-2, thus stabilizing the integrity of the mitochondrial outer membrane. Regarding ferroptosis, SA activates the SLC7A11-GPX4 pathway on one hand and reduces the expression of ferrous ions and LOX on the other, thereby decreasing lipid peroxidation (reductions in MDA and 4-HNE) and mitigating ferroptosis. The right side of the diagram illustrates pro-apoptotic effects of SA in the context of cancer. SA activates the generation of ROS, which promotes the nuclear transcription of p53, thereby activating the mitochondrial-dependent apoptosis pathway. Red circular and T-shaped arrows represent promotion and inhibition by SA, respectively. Dashed arrows indicate indirect effects. 4-HNE, 4-hydroxynonenal; APAF1, apoptotic protease activating factor 1; ATF6, activating transcription factor 6; BAX, BCL-2-associated X; BAK, BCL-2-antagonist/killer; BCL-2, B-cell lymphoma 2; CHOP, C/EBP homologous protein; CREB, cAMP response element-binding protein; Cys, cystine; Glu, glutamate; GPX4, glutathione peroxidase 4; GRP78, glucose-regulated protein 78; GSH, glutathione; LOX, lipoxygenase; MDA, malondialdehyde; PERK, protein kinase RNA-like endoplasmic reticulum kinase; ROS, reactive oxygen species; SA, syringic acid; SLC7A11, solute carrier family 7 member 11; Tf, Transferrin; TLR, toll-like receptor. Created in BioRender. Zhejun, Z. (2024) BioRender.com/g91x518.

SA plays a crucial role in mitigating oxidative stress and inflammation-related cellular pathological processes. Through multiple mechanisms, SA exerts protective effects against endoplasmic reticulum stress (ERS), apoptosis, and ferroptosis, which are key pathological processes aggravated by oxidative stress and inflammation, partially through modulating NRF2 and NF-κB signaling (Figure 5).

### 6.1 ERS

The ER is essential for cellular functions and survival, overseeing protein synthesis, folding, and trafficking (Oakes and Papa, 2015). ERS occurs when these critical processes are disrupted by pathological events, particularly oxidative stress and inflammation. To restore homeostasis, cells activate the unfolded protein response (UPR) pathway, which is regulated by three sensor proteins: activating transcription factor 6 (ATF6), inositol-requiring enzyme 1 (IRE1), and protein kinase RNA-like endoplasmic reticulum kinase (PERK), respectively (Bettigole and Glimcher, 2015; Chen and Cubillos-Ruiz, 2021). Under normal condition, glucose-regulated protein 78 (GRP78) binds to sensor proteins, keeping them inactive. Upon activation of the UPR, ATF6 migrates to the Golgi apparatus, becomes activated, and then enters the nucleus to increase the expression of proteins like GRP78, GRP94, and CHOP, aiming to restore ER function (Read and Schröder, 2021; Wiseman et al., 2022). However, prolonged PERK activation can induce CHOP expression, suppress the anti-apoptotic gene BCL-2, and promote apoptosis (Marciniak et al., 2004).

Demir et al. demonstrated that SA mitigates rat testicular damage induced by I/R by inhibiting the HMGB1/NF-κB axis and ERS, resulting in a dose-dependent reduction of oxidative stress, inflammation, and key ERS markers (including GRP78, ATF6, and CHOP) (Demir et al., 2023). Similar findings were observed in another study by the same group, showing SA’s protective effects against cisplatin-induced ovarian damage (Demir, 2023). Additionally, in the context of neurodegenerative diseases, SA covalently modify tau protein, inhibit the formation of amyloid fibrils, and alleviate neurotoxicity. Importantly, this study highlighted SA’s inhibitory effects on endoplasmic reticulum stress mediated by tau amyloid proteins (downregulation of ATF6) and apoptosis (downregulation of caspase-8 and caspase-3) (Li et al., 2021).

### 6.2 Apoptosis

Apoptosis is a precisely regulated form of cell death that occurs *via* two main pathways: extrinsic and intrinsic (Bertheloot et al., 2021). The intrinsic pathway is triggered by internal stress factors such as DNA damage or ERS, with the key event being the release of cytochrome c from the mitochondria (Ketelut-Carneiro and Fitzgerald, 2022). This pathway is regulated by the balance between pro- and anti-apoptotic proteins of the Bcl-2 family (Kesavardhana et al., 2020). In contract, the extrinsic pathway is initiated through the activation of membrane receptors, such as tumor necrosis factor receptor 1 (TNFR1) or toll-like receptors (TLRs) (Amarante-Mendes et al., 2018). Despite their distinct initiation mechanisms, both pathways converge on the activation of caspases, particularly caspase-3, which executes apoptosis.

Oxidative stress and inflammation contribute to apoptosis (Cameli et al., 2020; Kondylis et al., 2017). In such models, SA exerts its anti-apoptotic activity by upregulating BCL-2 and downregulating BAX, caspase-3, 8, and APAF1 (Akarsu et al., 2023; Li et al., 2021; Wang, F. et al., 2023; Zhang et al., 2020). Multiple pathway networks are involved in regulating the anti-apoptotic capabilities of SA. For example, in cardiomyocytes, SA activates the PI3K/AKT/GSK-3β signaling pathway to exert anti-apoptotic activities (Liu G. et al., 2020), while in RGC-5 cells, it protects against H_2_O_2_-induced apoptosis *via* the PI3K/AKT signaling pathway, suggesting its therapeutic role in diabetic retinopathy (Song et al., 2016). In a rat colitis model, SA alleviates oxidative stress, inflammation, and apoptosis by inhibiting the TLR4/NF-κB/iNOS pathway (Ghasemi-Dehnoo et al., 2024). Most studies on SA’s anti-apoptotic effects focus on the mitochondria-dependent intrinsic pathway. Mitochondrial dysfunction leads to the release of cytochrome C from the mitochondrial membrane into the cytoplasm, resulting in apoptosis (Nakagawa and Tayama, 2000). In the study by Helli et al., bisphenol A-induced mitochondrial dysfunction increases free radicals production, activating the mitochondrial apoptosis signaling pathway. SA’s anti-apoptotic effects were attributed to its potent antioxidant capacity, which scavenges free radicals (Helli et al., 2024). Additionally, SA promotes the phosphorylation of the upstream cAMP response element-binding protein (CREB) of Bcl-2, stabilizing the mitochondrial outer membrane and preventing cytochrome c release (Helli et al., 2024; Yan et al., 2022).

Inducing apoptosis is one of the fundamental principles of most current cancer treatments (Ouyang et al., 2012). In cancer cells, SA promotes apoptosis through the mitochondria-dependent pathway, including the downregulation BCL-2, and upregulation of P53, caspase-3, caspase-9, cytochrome C, BAX, and APAF1 (Abijeth and Ezhilarasan, 2020; Li et al., 2023; Velu et al., 2017). This cytotoxic effect often coincides with ROS accumulation (Gheena and Ezhilarasan, 2019; Yang et al., 2020). Structurally similar phenolic acids, such as ferulic and caffeic acids, also increase ROS levels in cancer cells, inducing cytotoxicity (Bao et al., 2023; Rajendra Prasad et al., 2011). ROS can activates P53 translocation to the nucleus and trigger the expression of pro-apoptotic genes (Srinivas et al., 2019). They can also directly affect mitochondria by reducing their membrane potential, promoting the release of cytochrome C and leading to apoptosis (Sinha et al., 2013). Therefore, SA’s ability to regulate ROS accumulation may be central to its anticancer activity.

The pro-oxidant effects of polyphenols in cancer cells are thought to be related to copper-mediated cellular DNA damage (Farhan and Rizvi, 2022). Malignant cells have a higher absolute copper content than non-malignant cells (Ge et al., 2022). In the presence of copper ions, polyphenolic metabolites can act as pro-oxidants. Specifically, plant polyphenols are believed to mobilize chromatin-bound copper, undergoing redox reactions that reduce Cu^2+^ to Cu^1+^, which is then re-oxidized by molecular oxygen to produce ROS. Although SA may promote oxidation in this manner, the extent of DNA damage depends on the orientation and number of hydroxyl groups, particularly galloyl groups (Farhan et al., 2015). SA has only one hydroxyl group at the 4-position, with methoxy groups at the 3- and 5-positions. Another possible hypothesis is that the two methoxy groups, as electron donors, push electron density towards the benzene ring through resonance, thereby increasing the electron density on the ring. This makes it easier for the ring to donate electrons to Cu^2+^, facilitating its reduction to Cu^1+^ and generating ROS. Additionally, the small molecular size of SA allows it to approach DNA closely, which is essential because hydroxyl radicals have a limited diffusion radius and must be generated near cellular DNA (Pryor, 1988). While SA exhibits significant pro-oxidant effects in cancer cells, the mechanisms underlying its dual anti-oxidant and pro-oxidant activities remain unclear, necessitating further research to validate these hypotheses.

### 6.3 Ferroptosis

Ferroptosis is a form of programmed cell death first identified in 2012, distinct from traditional modes of cell death such as apoptosis and autophagy. Its hallmark features include iron ions dependence and the excessive accumulation of lipid peroxides (Dixon et al., 2012). Current research suggests that ferroptosis is closely associated with oxidative stress (Xie et al., 2016). Iron is an essential element for many metabolic enzymes involved in the production of intracellular ROS. Through the non-enzymatic, iron-dependent Fenton reaction, iron can further catalyze the production of large amounts of ROS, thereby exacerbating the extent of lipid peroxidation and the generation of reactive compounds such as malondialdehyde (MDA) and 4-hydroxynonenal (4-HNE) (Conrad and Pratt, 2019). The excessive accumulation of lipid peroxides disrupts cell membrane integrity, ultimately triggering cell death.

SA has been shown to significantly reduces lipid peroxidation markers, potentially through modulation of the solute carrier family 7 member 11 (SLC7A11)/glutathione peroxidase 4 (GPX4) axis. SLC7A11 is a critical cystine-glutamate exchanger that constitutes part of system x_c_^−^. By regulating the import of cystine (one of the essential precursors for GSH synthesis), SLC7A11 directly influences the synthesis of GSH (Chen et al., 2021; Liu M.R. et al., 2021). As the most abundant antioxidant in mammalian cells, GSH not only scavenges ROS but also acts as a key substrate for GPX4. GPX4 utilizes GSH to convert lipid peroxides into non-toxic molecules, thereby halting the progression of lipid peroxidation reactions and contributing to the cell’s resilience against oxidative stress. Ferroptosis has been implicated in various pathological conditions, including I/R-induced organ damage (Stockwell et al., 2017). In C2C12 subjected to hypoxia/reoxygenation (H/R), demonstrated that SA can upregulate GPX4 and SLC7A11, while reducing intracellular Fe^2+^ levels and lipid peroxidation (Wang F. et al., 2023). Additionally, beyond GPX4, other key regulators of Ferroptosis, such as ferroportin and HO-1, are transcriptional targets of nuclear factor erythroid 2-related factor 2 (NRF2) (Kerins and Ooi, 2018). In fact, many proteins and enzymes responsible for preventing lipid peroxidation and thereby triggering ferroptosis are target genes of NRF2 (Dodson et al., 2019). Notably, NRF2 inhibitors have been explored as potential inducers of ferroptosis-mediated cancer cell death (Roh et al., 2017; Sun et al., 2016). Given the significant effects of SA on modulating NRF2 activity, while direct evidence linking SA to ferroptosis remains limited, there is a strong rationale to investigate this association further.

## 7 Pharmacokinetics

Ideal pharmacokinetic properties (PPs) are a prerequisite for the clinical application of a botanical drug. Understanding a botanical drug’s PPs helps elucidate its absorption, distribution, metabolism, and excretion (ADME) processes (Bai et al., 2021). A series of studies have assessed the bioavailability and tissue distribution of SA administered orally in rats and mice (Liu et al., 2019; Sun et al., 2020; Sun et al., 2021; Sun et al., 2018). Following a single oral administration of SA in rats, the pharmacokinetic curve shows a rapid and sharp contour, reaching the maximum plasma concentration within 5–15 min, and then swiftly diminishing from the rat plasma. The maximum plasma concentration (C_max_), half-life (T_1/2_), the area under the curve (AUC), and the mean residence time (MRT) ranges between 4.49 and 9.86 μg/mL, 14.29–25.36 min, 200.34–667.22 μg/mL*h, and 34.8–51 min, respectively. Notably, the absolute bioavailability of SA in rabbits was determined to be 86.27%, as calculated by comparing AUC following intraperitoneal administration to that after intravenous injection (Liu et al., 2003). Despite its high bioavailability in rabbits, SA exhibits poor absorption and rapid elimination when taken orally, contributing to its short T1/2 and relatively low bioactivity (Sun et al., 2020). The biodistribution of SA after a single oral dose in mice was also assessed. SA was widely distributed to most organs within 15 min, with the most significant accumulation in the kidneys, followed by the liver, lungs, spleen, and heart. In addition, SA also has the capability to cross the blood-brain barrier (BBB). By 120 min, SA had disappeared from most organs. SA primarily distributes to well-perfused tissues such as the liver and kidneys, dependent on blood flow and organ perfusion rates. The significant distribution of SA in the kidneys suggests its clearance mainly through metabolic pathways, while accumulation in the liver reflects a process of passive capture, possibly indicating that part of the drug did not effectively participate in physiological activities. Although direct bioavailability values have not been studied, the rapid elimination rate of the drug, passive accumulation in the liver, and clearance mechanisms through the kidneys could collectively explain the low bioavailability of SA.

SA’s PPs in medicinal plants show significant differences compared to those of the purified metabolite, manifested by varying degrees of increase in AUC, C_max_, MRT, and T_1/2_ (Table 3). This is attributed to the synergistic effects of various constituents within the extract. Even in scenarios where SA constitutes a minimal proportion of the plant extract, as is the case with *Cynanchum auriculatum*, where SA accounts for merely 0.01%, the *in vivo* half-life of SA within rats notably extends to 34.52 h (Sun et al., 2023). It is noteworthy that the study also evaluated the PPs of SA under conditions of functional dyspepsia, finding that the disease state did not significantly impact the PPs of SA. This may imply that the absorption process of SA exhibits a certain degree of robustness, or that its absorption mechanism may rely on pathways not directly affected by the state of the digestive system. It is believed that phenolic metabolites are absorbed mainly through passive diffusion mechanisms or *via* specific carriers present in the intestine, such as P-glycoprotein and the sodium-glucose linked transporter 1(SGLT1) cotransporter (Lewandowska et al., 2013). However, research on the PPs of SA *in vivo* under disease conditions remains limited. A deeper understanding of the absorption and metabolism mechanisms of SA in specific health conditions still requires further experimentation and observation. In addition to the synergistic effects between metabolites, existing research has employed various drug delivery system optimization strategies to enhance the oral bioavailability of SA. These include the use of TPGS/F127/F68 mixed polymer microcapsules (Sun et al., 2020), liposomes (Sun et al., 2018), TPGS liposomes (Liu et al., 2019), and self-microemulsifying drug delivery systems (Sun et al., 2021), all of which have been shown to at least double the relative bioavailability.

**TABLE 3:** Pharmacokinetics of SA *in vivo*.

Source/Formulation	Doses (g/kg)	Animal	Effects on PPs of SA	References
*Cynanchum auriculatum* Royle ex Wight (root)	1	SD rats	↓C_max_, ↓AUC; ↑T_1/2_	Sun et al. (2023)
*Prunus mume* (Siebold) Siebold & Zucc. (fruit)	5	SD rats	↑AUC, ↑C_max_, ↑MRT, ↑T_1/2_	Zhu et al. (2022)
*Echinacea purpurea* (L.) Moench (flower)	10	SD rats	↑AUC, ↑C_max_, ↑MRT, ↑T_1/2_, ↑T_max_	Du et al. (2017)
*Mahonia fortunei* *Berberis fortunei* (Lindl.) Fedde (stem)	5	SD rats	↑AUC, ↑C_max_, ↑MRT, ↑T_1/2_	Liu, L. et al. (2020)
TPGS/F127/F68 mixed polymeric micelles	0.025	SD rats	↑AUC, ↑C_max_, ↑MRT, ↑T_1/2_	Sun et al. (2020)
TPGS liposome	0.025	SD rats	↑AUC, ↑C_max_, ↑MRT, ↑T_1/2_	Liu et al. (2019)
Self-microemulsifying drug delivery system	0.025	SD rats	↑AUC, ↑C_max_, ↑MRT, ↑T_1/2_	Sun et al. (2021)
Liposome	0.025	SD rats	↑AUC, ↑C_max_, ↑MRT, ↑T1/2	Sun et al. (2018)

Notes: The sources listed in the table refer to specific parts of medicinal plants, while formulations pertain to drug delivery systems. Scientific names of all plant species were checked and standardized according to the Medicinal Plant Names Services (MPNS) database (http://mpns.kew.org/mpns-portal/). The effects on PPs are compared with those of orally administered SA as discussed in the text.

Abbreviations: AUC, area under the curve; C_max_, maximum plasma concentration; MRT, mean residence time; T_1/2_, half-life; T_max_, time to reach maximum plasma concentration.

## 8 Toxicity and safety assessment

SA is generally considered non-toxic to animals at therapeutic doses (Srinivasulu et al., 2018). A study on the subacute toxicity of SA by Mirza and Panchal, wherein Wistar rats received 1,000 mg/kg/day *via* oral gavage for 14 days, without significant adverse effects on body weight, food intake, erythropoiesis, leukopoiesis, or the internal organs including the liver, heart, kidneys, pancreas, hippocampus, and sciatic nerve (Mirza and Panchal, 2019). These findings suggest SA is safe within a limited timeframe. However, no clinical studies have been conducted on humans.

An *ex vivo* study involving 44 individuals, including healthy donors and patients with acute myeloid leukemia, demonstrated that treating peripheral blood mononuclear cells with 10 µM SA for 1 h did not show any signs of cellular damage (Haddadi et al., 2023). Although “Occupational hepatotoxins - Secondary hepatotoxins” describes the potential for a toxic effect in the occupational setting based on human ingestion or animal experimentation (https://pubchem.ncbi.nlm.nih.gov/compound/Syringic-acid), it is important to note that these risks are associated with specific conditions, such as prolonged exposure or high concentrations. Therefore, while passive accumulation of SA in the liver could theoretically contribute to its potential as an occupational hepatotoxin, there is currently no evidence to suggest that such risks are relevant in clinical use, particularly given the therapeutic doses and short exposure times typically involved. According to hazard classification, SA can cause skin, eye, and respiratory irritation (https://pubchem.ncbi.nlm.nih.gov/compound/Syringic-acid). These classifications underline the need for caution when handling SA, especially in occupational environments where exposure risk is higher.

## 9 Discussion

This review highlights the broad-spectrum pharmacological activities of SA, emphasizing its potent antioxidative and anti-inflammatory properties. As elucidated, SA exerts its effects through intricate mechanisms, modulating critical cellular pathways including NRF2, NF-κB, and PI3K/AKT, which play pivotal roles in cellular defense against oxidative stress and inflammation. By influencing these pathways, SA mitigates ERS, apoptosis, and ferroptosis, thereby restoring cellular pathological processes to their normal state. Evidence suggests that SA’s anti-inflammatory and antioxidant effects are primarily mediated through NRF2 activation and NF-κB inhibition.

### 9.1 Research gap

This review focuses on the antioxidant and anti-inflammatory activities of SA. Despite its promising properties, current research is insufficient, with several critical issues unresolved. Most studies lack systematic *in vivo* validation, appropriate positive controls, and mechanistic depth. The mechanisms underlying SA’s antioxidant activity remain inadequately elucidated, posing a significant challenge. While SA’s significant anti-inflammatory activity is better supported, the causal relationship between its antioxidant and anti-inflammatory activities is not well-established. Determining whether SA’s primary action is antioxidant or anti-inflammatory is imperative for future research. Most *in vivo* studies heavily rely on rats for basic mechanism validation, limiting research depth. Few studies have elucidated the minimum effective antioxidant concentration of SA *in vivo*. Significant differences exist in the pharmacokinetics of SA derived from medicinal plants compared to the purified metabolite, and its pharmacokinetics in humans remain largely unexplored. Comparative efficacy studies relative to other antioxidants and anti-inflammatory agents are scarce. Furthermore, the long-term effects and safety profile of SA, particularly concerning potential hepatotoxicity with prolonged administration, are insufficiently explored.

There also remains a lack of direct target engagement studies, such as protein–ligand interaction assays (e.g., surface plasmon resonance, thermal shift assays, or cellular target validation), to confirm the molecular targets of SA. Structure–activity relationship (SAR) analyses have been insufficiently addressed. Moreover, few studies have directly compared the efficacy of SA with established clinical antioxidants or anti-inflammatory drugs, making it difficult to contextualize its therapeutic relevance.

### 9.2 Translational potential and challenges in clinical settings

While SA demonstrates substantial antioxidant and anti-inflammatory potential in preclinical studies, its translation into clinical settings faces several challenges. SA’s pharmacokinetics, particularly its limited bioavailability, remains a significant hurdle for clinical application. The current research has shown that SA’s bioavailability can be improved *via* intraperitoneal injection to bypass first-pass metabolism; however, this raises concerns about potential hepatic overexposure and associated toxicity. Optimizing drug delivery methods, such as nanoparticles, liposomes, or polymeric micelles, could improve its therapeutic potential, ensuring better tissue distribution and reducing the risk of toxicity.

Another key challenge is the variability in the pharmacokinetics of SA derived from different sources. The differences in ADME between plant-derived SA and the purified metabolite need to be further studied to understand how these factors may influence clinical outcomes. Additionally, the long-term safety and efficacy of SA, especially with regard to its potential hepatotoxicity after prolonged use, must be carefully evaluated in clinical trials. Furthermore, while SA has demonstrated significant effects in animal models of oxidative stress and inflammation, translating these results to human trials will require robust evidence of its effectiveness and safety at therapeutic doses. There is a need for well-designed clinical studies to establish appropriate dosing regimens and assess the metabolite’s interactions with other therapeutic agents. Combining SA with existing drugs in therapies could also improve treatment outcomes while minimizing side effects.

Future research should prioritize clinical trials to evaluate SA’s efficacy and safety in humans, focusing on its pharmacokinetics, dosage optimization, and potential for synergistic effects with other agents. Mechanistic insights into how SA modulates key signaling pathways such as NRF2, NF-κB, and PI3K/AKT will also be crucial for understanding its therapeutic potential in clinical settings.

### 9.3 Limitations

Several limitations of this review should be acknowledged. Although this work provides a comprehensive overview of the antioxidant and anti-inflammatory properties of SA, the majority of the included studies are preclinical, with considerable heterogeneity in experimental design, dosage, and model systems. A portion of the included literature is based on *in vitro* experiments, which, while mechanistically informative, may not fully capture the metabolite’s effects under physiological conditions. Moreover, as SA is a polyphenolic compound, concerns have been raised regarding its potential classification as a pan-assay interference compound (PAINS), known to cause false-positive results in high-throughput or cell-based assays. Nevertheless, structural alert screening using both the ZINC database (https://zinc15.docking.org/) and SwissADME (http://www.swissadme.ch/) revealed no PAINS-associated structural features in SA. While this supports its potential specificity, caution is still warranted when interpreting *in vitro* findings involving polyphenols.

In addition, potential publication bias cannot be excluded, given that the number of publications highlighting the anti-inflammatory effects of syringic acid substantially exceeds those investigating its antioxidant activity. Studies published in non-English languages or non-indexed journals may have been overlooked. Finally, due to the narrative nature of this review, quantitative comparisons, meta-analyses, or statistical effect size estimations were not performed.

## 10 Conclusion

In summary, SA has demonstrated considerable antioxidant and anti-inflammatory pharmacological activities, highlighting its potential as a therapeutic agent. However, further research is needed to clarify its mechanisms of action, optimize its pharmacokinetics, and evaluate its long-term safety and efficacy in clinical settings. By addressing these challenges and focusing on translational research, SA could emerge as a valuable therapeutic option for conditions associated with oxidative stress and inflammation.

## Glossary

4-HNE: 4-hydroxynonenal
AKT: Protein kinase B
APAF1: Apoptotic protease activating factor 1
ARE: Antioxidant response element
ATF6: Activating transcription factor 6
BAX: BCL-2-associated X
BAK: BCL-2-antagonist/killer
BCL-2: B-cell lymphoma 2
BVR: Biliverdin reductase
CAT: catalase
CHOP: C/EBP homologous protein
CO: Carbon monoxide
COX-2: Cyclooxygenase-2
CREB: cAMP response element-binding protein
Crm1: Chromosomal region maintenance 1
Cul3: cullin-3
Cys: cystine
ERS: Endoplasmic reticulum stress
Fyn: Fyn tyrosine kinase
GA: Gallic acid
Glu: Glutamate
GPx: Glutathione peroxidase
GRP78: Glucose-regulated protein 78
GSH: Glutathione
GSK-3β: Glycogen synthase kinase 3β
GST: Glutathione S-transferase
HMGB1: High mobility group box 1
HO-1: Heme oxygenase-1
H/R: Hypoxia/reoxygenation
IFN-γ: Interferon-γ
IKK: IκB kinase
IκB: Inhibitor κB
IL: Interleukin
iNOS: Inducible nitric oxide synthase
I/R: Ischemia/reperfusion
IRE1: Inositol-requiring enzyme 1
KEAP1: Cytoplasmic kelch-like epichlorohydrin-associated protein 1
L-NAME: Nω-nitro-L-arginine methyl ester
LOX: Lipoxygenase
MDA: Malondialdehyde
MPO: Myeloperoxidase
MyD88: Myeloid differentiation primary response 88
NADPH: Nicotinamide adenine dinucleotide phosphate
NDMA: N-nitrosodimethylamine
NF-κB: Nuclear factor-kappa B
NO: Nitric oxide
NOS: NO synthase
NOX: NADPH oxidase
NQO1: NADPH quinone oxidoreductase enzyme
NRF2: Nuclear factor erythroid 2-related factor 2
OGD/R: Oxygen-glucose deprivation/reoxygenation
PERK: Protein kinase RNA-like endoplasmic reticulum kinase
PGE2: Prostaglandin E2
PI3K: Phosphatidylinositol-4,5-bisphosphate 3-kinase
PIP2: Phosphatidylinositol 4,5-bisphosphate
PIP3: Phosphatidylinositol 3,4,5-trisphosphate
PPs: Pharmacokinetic properties
RBX1: Ring-box 1
RNS: Reactive nitrogen species
ROS: Reactive oxygen species
SA: Syringic acid
SLC7A11: Solute carrier family 7 member 11
sMAF: small musculoaponeurotic fibrosarcoma
SOD: Superoxide dismutase
TAS: Total antioxidant status
TCM: Traditional Chinese medicine
Tf: Transferrin
TLR: Toll-like receptor
TNF-α: Tumor necrosis factor-α
TNFR1: Tumor necrosis factor receptor 1
TOS: Total oxidant status
TRAF6: Tumor necrosis factor receptor-associated factor 6
UPR: Unfolded protein response.
